# Supplementary material for: A Global Screen for Assembly State Changes of the Mitotic Proteome by SEC-SWATH-MS
Source: Cell Syst. 2020 Feb 26;10(2):133–155.e6. doi: 10.1016/j.cels.2020.01.001 (PMC7042714; doi:10.1016/j.cels.2020.01.001)

Q9UM00 | TMCO1_HUMAN | TMCO1 TMCC4 PNAS−10 PNAS−136 UNQ151/PRO177
Monomer MW [kDa]: 21.175 Monomer expected elution fraction: 54

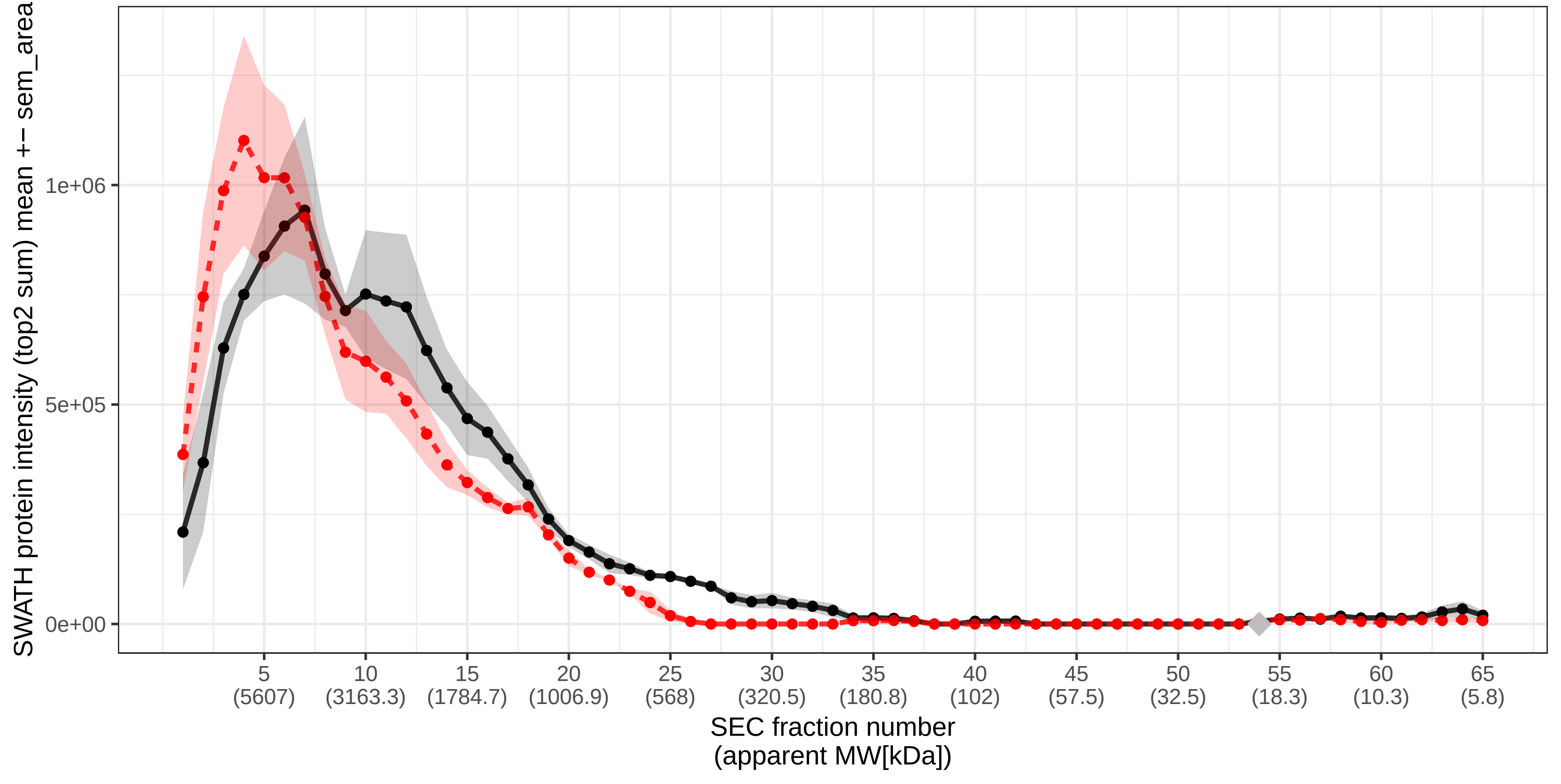

Supplement: Data S1. SEC-SWATH-MS Protein Chromatograms, Related to Figure 1 [file mmc6.zip › SECchrom_Q9UM00_TMCO1_HUMAN_TMCO1_TMCC4_PNAS-10_PNAS-136_UN.pdf]
